# Social psychology: Predicting other people’s attention to understand their mind

**DOI:** 10.1038/s44271-023-00037-6

**Published:** 2023-11-28

**Authors:** Margot Gueguen, Patricia L. Lockwood

**Affiliations:** Centre for Human Brain Health, University of Birmingham, Birmingham, UK

**Keywords:** Psychology

## Abstract

Humans are highly social beings who are interested in what others are saying, thinking, and doing. A recent study in the Proceedings of the National Academy of Sciences finds that we can easily tell whether a person’s pattern of attention is natural or artificially manipulated.

**Figure:**
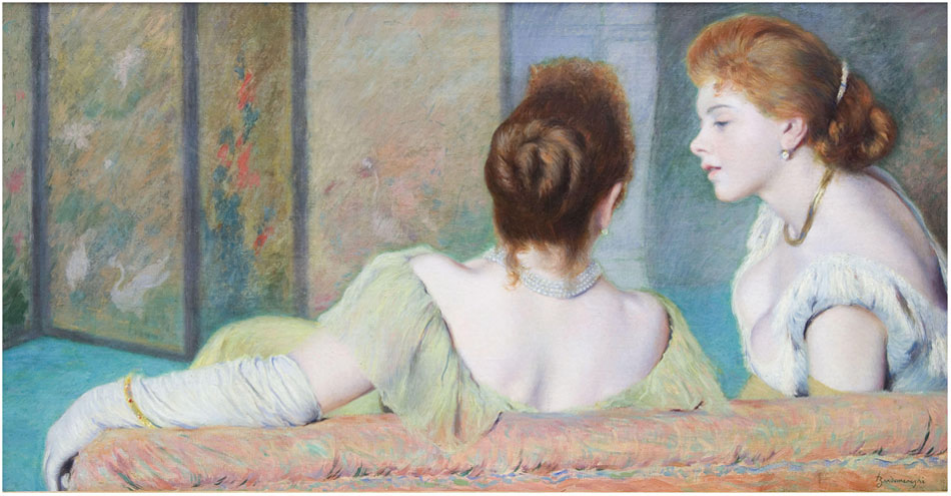
On the couch, 1885–1890, Federico Zandomeneghi. Photo: Alamy.

In our day-to-day lives, we tend to spend a lot of time figuring out what’s going on in other people’s minds. This is known as “theory of mind” and may be unique to humans. Some disorders can disrupt this ability, resulting in impaired social behaviour. Previous research has focused on how we pay attention to where other people are looking, but it did not consider how we use this information to predict their actions or thoughts.

A new study by Ziman and colleagues at Princeton University argues that rather than simply monitoring where others gaze, humans use this information to understand their minds^1^. The authors asked 118 participants across 8 experiments to watch movies of a bright spot navigating around a scene based on the gaze patterns of prior participants. On each trial, participants were asked to differentiate between a natural gaze sequence and a sequence which was artificially manipulated. Depending on the experiment, the artificial sequence may have been scrambled, backwards, mirrored, or on the wrong background image. Natural and artificial sequences where the bright spot navigated around blank backgrounds were also compared. In nearly all cases, participants could distinguish between real and artificial movements. They could even distinguish between the gaze patterns of two different individuals.

This ability to understand others based on where they look is important. It fits with recent work suggesting that there may be specific brain areas for monitoring other people’s motivation. It also aligns with studies in non-human animals showing a strong neural response when two individuals make eye contact during social interactions. The next step is to test how differences in this ability relate to differences in behaviour, such as when people engage in real-life social interaction and need to understand others’ thoughts and motivations. Answering these questions will be essential for helping those who struggle with theory of mind and solving complex global challenges that rely on understanding and cooperating with other people.
